# Evaluation of Absolute Neutrophil, Lymphocyte and Platelet Count and Their Ratios as Predictors of Thrombotic Risk in Patients with Prefibrotic and Overt Myelofibrosis

**DOI:** 10.3390/life14040523

**Published:** 2024-04-17

**Authors:** Marko Lucijanic, Ivan Krecak, Ena Soric, Anica Sabljic, Davor Galusic, Hrvoje Holik, Vlatka Perisa, Martina Moric Peric, Ivan Zekanovic, Josipa Budimir, Rajko Kusec

**Affiliations:** 1 Hematology Department, University Hospital Dubrava, Av. Gojka Suska 6, 10000 Zagreb, Croatia; 2 School of Medicine, University of Zagreb, Ul. Salata 3, 10000 Zagreb, Croatia; 3 Department of Internal Medicine, General Hospital Sibenik, Ul. Stjepana Radica 83, 22000 Sibenik, Croatia; 4 School of Medicine, University of Rijeka, Ul. Brace Branchetta 20/1, 51000 Rijeka, Croatia; 5 Sibenik University of Applied Science, Trg Andrije Hebranga 11, 22000 Sibenik, Croatia; 6 Department of Hematology, University Hospital of Split, Soltanska ul. 1, 21000 Split, Croatia; 7 School of Medicine, University of Split, Soltanska ul. 2, 21000 Split, Croatia; 8 Department of Internal Medicine, “Dr. Josip Bencevic” General Hospital, Ul. Andrije Stampara, 35000 Slavonski Brod, Croatia; 9 Faculty of Medicine, University of Osijek, Ul. Josipa Huttlera 4, 31000 Osijek, Croatia; 10 Department of Hematology, Osijek University Hospital, Ul. Josipa Huttlera 4, 31000 Osijek, Croatia; 11 Department of Internal Medicine, General Hospital Zadar, Ul. Boze Pericica 5, 23000 Zadar, Croatia

**Keywords:** complete blood count, myeloproliferative neoplasms, polycythemia vera, essential thrombocythemia, primary myelofibrosis

## Abstract

Aim: To investigate the prognostic contribution of absolute neutrophil (ANC), lymphocyte (ALC), platelet count and their ratios, neutrophil–lymphocyte ratio (NLR) and platelet–lymphocyte ratio (PLR), to thrombotic risk in patients with prefibrotic and overt fibrotic myelofibrosis (MF). Methods: We retrospectively analyzed a cohort of 256 patients with prefibrotic (85 patients) and overt fibrotic MF (171 patients) treated in six Croatian hematological centers. Results: Prefibrotic compared to overt fibrotic MF patients presented with significantly higher ALC, platelet count and PLR, and experienced longer time to thrombosis (TTT). Among prefibrotic patients, ANC > 8.33 × 10^9^/L (HR 13.08, *p* = 0.036), ALC > 2.58 × 10^9^/L (HR 20.63, *p* = 0.049) and platelet count > 752 × 10^9^/L (HR 10.5, *p* = 0.043) remained independently associated with shorter TTT. Among overt fibrotic patients, ANC > 8.8 × 10^9^/L (HR 4.49, *p* = 0.004), ALC ≤ 1.43 × 10^9^/L (HR 4.15, *p* = 0.003), platelet count ≤ 385 × 10^9^/L (HR 4.68, *p* = 0.004) and chronic kidney disease (HR 9.07, *p* < 0.001) remained independently associated with shorter TTT. Conclusions: Prognostic properties of ANC, ALC and platelet count are mutually independent and exceed those of NLR and PLR regarding thrombotic risk stratification. ALC and platelet count associate in opposite directions with thrombotic risk in prefibrotic and overt fibrotic MF patients.

## 1. Introduction

BCR::ABL negative myeloproliferative neoplasms (MPNs) are driven by constitutional JAK-STAT (Janus kinase-signal transducer and activator of transcription) activation in hematopoietic stem cell progenitors [1], resulting in increased production of mature blood cells and various degrees of cytoses and cytopenias [2]. Underlying mutations in JAK2, CALR and MPL genes, which are considered to be mutually exclusive, may differently affect clinical presentation of MPN patients. Three classical clinical presentations are recognized, namely, polycythemia vera (PV), characterized by trilineage proliferation and increase in circulating red blood cells; essential thrombocythemia (ET), characterized by isolated increase in platelet count; and primary myelofibrosis (PMF), characterized by anemia, splenomegaly and constitutional symptoms. However, both PV and ET may progress to the secondary myelofibrotic stage (secondary myelofibrosis, SMF) that is clinically like fully developed PMF, and PMF is preceded by the prefibrotic stage termed prefibrotic PMF, which is an ET-like disease from which it can be accurately discriminated by insight into the bone marrow (BM) morphology. All three classic MPN subsets are highly burdened by high risk of arterial and venous thromboses [3,4,5]. Thrombotic risk guides PV and ET stratification and treatment [2,6], whereas thrombotic risk in PMF and SMF patients is typically insufficiently recognized since these patients are usually evaluated and managed through the prism of debilitating constitutional symptoms and risk of death [7]. Among myelofibrosis (MF) patients, further differences in thrombotic risk may exist [8], which are probably harbored from the previous non-fibrotic disease stages. Inability to provide proper thromboprophylaxis or cytoreductive therapy in MF patients with cytopenias may also play a role [9,10].

Pathogenesis of thrombotic events in MF, as well as in MPN in general, is complex and not only related to MPN clonal characteristics (peripheral counts, genetic features, etc.) [11,12,13,14], but is also sustained by chronic inflammation, immune dysregulation [15,16] and chronic metabolic comorbidities [17]. The stage of overt fibrotic MF represents years of prior subclinical clonal hematopoiesis or established MPN [18,19,20] and, thus, long-term exposure to fibrotic and inflammatory stimuli affecting not only bone marrow but also vasculature [21,22], kidneys [23], osteo-muscular [24] and other organ systems [25,26]. For the even higher complexity, the MPN disease clone may produce not only prothrombotic, but also cardioprotective cytokines like heat shock protein 27 (HSP27/HSPB1) [27,28], whose higher expression seems to be associated with improved survival [27].

Components of the complete blood count (CBC), the first and foremost tool in the hematologist armamentarium, have been shown to bear clinical and prognostic information that goes beyond diagnostic recognition of hematological entities [29]. Although absolute white blood cell count (WBC) and percentage of circulatory blasts are recognized as important predictor of disease prognosis among PMF patients [30], in the recent years, other WBC subsets (neutrophils, lymphocytes, monocytes and basophils) and their ratios (neutrophil-to-lymphocyte ratio, NLR, and platelet-to-lymphocyte ratio, PLR) have gained recognition as contributors to prognosis in patients with PMF and other MPNs [31,32,33,34]. ANC, ALC and age have recently been incorporated into the triple A (AAA) prognostic score [35], shown to predict mortality in ET, PV and MF [35,36,37], and thrombotic risk in PV patients [36]. The utility of components of the CBC to provide prognostic information regarding thrombotic risk in patients with MF is unknown and has not been investigated so far. Thus, in this study, we aimed to systematically investigate clinical associations and prognostic contribution of absolute neutrophil (ANC), lymphocyte (ALC), platelet count and their ratios, NLR and PLR, in patients with prefibrotic and overt fibrotic MF.

## 2. Materials and Methods

### 2.1. Patients and the Methodology

We retrospectively analyzed a cohort of 256 patients with prefibrotic (85 patients) and overt fibrotic MF (171 patients) treated in six Croatian hematological centers in the period from 2004 to 2023. Diagnoses were reassessed by the 2016 World Health Organization (WHO) criteria for prefibrotic and overt fibrotic PMF [38], and by the 2008 International Working Group for Myelofibrosis Research and Treatment (IWG-MRT) criteria for post-PV and post-ET SMF [19]. The degree of BM fibrosis was classified using the European consensus criteria [39]. Severity of disease was categorized by The Dynamic International Prognostic Scoring System (DIPSS) for PMF [30] and MYelofibrosis SECondary to PV and ET prognostic model (Mysec-PM) for SMF patients [40]. Comorbidities were assessed as individual entities and cumulatively through the Charlson comorbidity index. Baseline clinical characteristics and laboratory parameters were analyzed, and results are presented from the perspective of ANC, ALC, platelet count, NLR and PLR, respectively. Analyses were performed separately for prefibrotic and overt fibrotic MF patients.

Time to thrombosis (TTT) was evaluated from the time of diagnosis/referral to the first arterial or venous thrombotic event. Arterial thrombotic events that were considered were myocardial infarction, cerebrovascular infarction and peripheral arterial thromboses. Venous thrombotic events that were considered were deep venous thrombosis, pulmonary embolism, cerebral and splanchnic venous thromboses. Due to statistical power concerns associated with a low number of patients and events in particular subgroups, thrombotic events were evaluated as a composite arterial and venous thrombosis endpoint. 

The study was approved by the Institutional Review Boards of the University Hospital Dubrava (2020/0306-05), the University Hospital Center Split (2181-147-01/06/M.S.-19-3), the University Hospital Center Osijek (R2-1060/2020), the General Hospital Zadar (02-2025/20-6/20), the General Hospital of Sibenik-Knin County (01-3618/1-20) and the Dr. Josip Bencevic General Hospital (04000000/20-37). All procedures followed were in accordance with the ethical standards of the responsible committee on human experimentation (institutional and national) and with the Helsinki Declaration of 1975, as revised in 2008. 

### 2.2. Statistical Methods

Numerical variables were evaluated for the normality of distribution using the Shapiro–Wilks test. Due to the non-normal distribution of majority of the investigated numerical variables, they were all presented as medians and interquartile ratios (IQR). Categorical variables were represented as ratios and percentages. The Kruskal–Wallis test and the Spearman rank correlation were used to compare investigated CBC indices with clinical characteristics. The receiver operating characteristic (ROC) curve analysis was used to recognize and evaluate optimized cut-off values (with the highest Youden index) of hematological indices for thrombotic events prediction. Time to event analyses were based on the Kaplan–Meier method. Time to event curves were compared between groups using the log-rank test. Multivariate time to event analyses were performed using the Cox regression analysis. *p* values < 0.05 were considered statistically significant. Screening of time to event associations was performed using the custom-made Microsoft Excel workbook [41]. All presented analyses were performed using the MedCalc statistical program version 22.017 (MedCalc Software Ltd., Ostend, Belgium).

## 3. Results

### 3.1. Patients’ Characteristics

We analyzed a cohort of 256 patients with MF. Among them, there were 85 patients with prefibrotic PMF, 94 with overt PMF, 39 with post-PV SMF and 38 with post-ET SMF. There were 155 (60.5%) male patients. The median age was 68 years, IQR (60–75). Patients’ characteristics for prefibrotic and overt fibrotic MF patients are shown in Table 1 and Table 2, respectively.

The median ANC was 7.4 × 10^9^/L, IQR (3.92–12.73), and it did not significantly differ between prefibrotic and overt fibrotic MF patients (median 7.5 vs. 7.3, *p* = 0.367). The median ALC was 1.5 × 10^9^/L, IQR (1.1–2.26), and prefibrotic patients presented with significantly higher ALC in comparison to overt fibrotic MF patients (median 1.77 vs. 1.4, *p* = 0.002). The median platelet count was 363.5 × 10^9^/L, IQR (203–595), and prefibrotic patients presented with significantly higher platelet count in comparison to overt fibrotic MF patients (median 550 vs. 300, *p* < 0.001). 

The median NLR value was 4.5, IQR (2.6–7.67), and it did not significantly differ between prefibrotic and overt fibrotic MF patients (median 4.26 vs. 4.5, *p* = 0.596). The median PLR value was 226.4, IQR (112.49–407.86), and prefibrotic patients presented with significantly higher PLR values in comparison to overt fibrotic MF patients (median 344.19 vs. 200, *p* = 0.021).

Clinical associations of ANC, ALC and platelet count are shown in Table 1 and Table 2, respectively. Among prefibrotic PMF patients, higher ANC was significantly associated with older age, JAK2 mutation, absence of CALR mutation, present constitutional symptoms, higher WBC, higher ALC, monocyte and basophil count, higher lactate dehydrogenase (LDH), serum uric acid, Charlson comorbidity index, presence of chronic kidney disease and use of cytoreductive therapy (*p* < 0.05 for all analyses). Higher ALC in prefibrotic PMF patients was associated with female sex, higher WBC, ANC, monocyte and basophil count (*p* < 0.05 for all analyses). Higher platelet count in prefibrotic PMF patients was significantly associated with male sex, higher grade of BM fibrosis, absence of overt MF clinical features (constitutional symptoms, transfusion dependency, massive splenomegaly, larger spleen size, circulatory blasts), lower C-reactive protein (CRP) and use of cytoreductive therapy (*p* < 0.05 for all analyses). Among overt MF patients, higher ANC was significantly associated with JAK2 mutation, higher WBC, higher ALC, monocyte and basophil count, hemoglobin, platelets, LDH, serum uric acid and use of cytoreductive therapy (*p* < 0.05 for all analyses). Higher ALC in overt fibrotic patients was significantly associated with absence of massive splenomegaly, higher WBC, presence of circulatory blasts, higher ANC, monocyte and basophil count, higher LDH and higher serum uric acid (*p* < 0.05 for all analyses). Higher platelet count in overt fibrotic patients was significantly associated with male sex, lower grade of bone marrow fibrosis, absence of constitutional symptoms, lower spleen size, higher WBC, higher monocyte and basophil count, hemoglobin, lower CRP, higher serum uric acid, presence of chronic kidney disease and hyperlipoproteinemia, absence of diabetes mellitus, use of cytoreductive therapy, lower DIPSS among PMF and lower Mysec-PM among SMF patients (*p* < 0.05 for all analyses).

A total of 48 (18.75%) patients experienced thrombotic events prior to or at the time of diagnosis. After median follow-up time of 44 months, a total of 33 patients experienced thrombotic event in follow-up (24 arterial and 10 venous thromboses). Median TTT was not reached, neither for composite, arterial nor venous thrombotic events. TTT significantly differed between prefibrotic and overt fibrotic MF patients, with higher risk of thrombosis associated with overt fibrotic status (HR 2.04, *p* = 0.046), which was driven by arterial thrombotic events (HR 2.8, *p* = 0.015) but no significant difference was present regarding venous thrombotic events (*p* = 0.455). Freedom from thrombosis rates were 90.5% and 82.8% at 5-year and 87.4% and 60.6% at 10-year milestones for prefibrotic and overt fibrotic MF patients, respectively.

Associations with thrombotic events were further evaluated for prefibrotic and overt fibrotic patients separately.

### 3.2. Relationship of Absolute Neutrophil, Absolute Lymphocyte, Platelet Count, Neutrophil-to-Lymphocyte and Platelet-to-Lymphocyte Ratios with Future Risk of Thrombosis in Prefibrotic PMF Patients

Using the ROC curves analysis, specific cut-off points with best predictive properties for thrombotic risk prognostication for the context of prefibrotic PMF were defined for ANC, ALC, platelets, NLR and PLR and are presented in Table 3. 

For prognostication of composite thrombotic endpoint, arterial and venous thromboses, respectively, the ROC curve-defined optimized cut-off values for ANC (×10^9^/L) were >8.33, >8.33, >14.19, respectively, for ALC (×10^9^/L) were >2.58, >4, and >2.6, respectively, and for platelet count (×10^9^/L) were >752, >574, and no adequate cut-off, respectively. The ROC curve-defined cut-off values for NLR were >6.33, no adequate cut-off, and >6.33, respectively, and for PLR were >498, >498, and no adequate cut-off, respectively.

**Figure 1:**
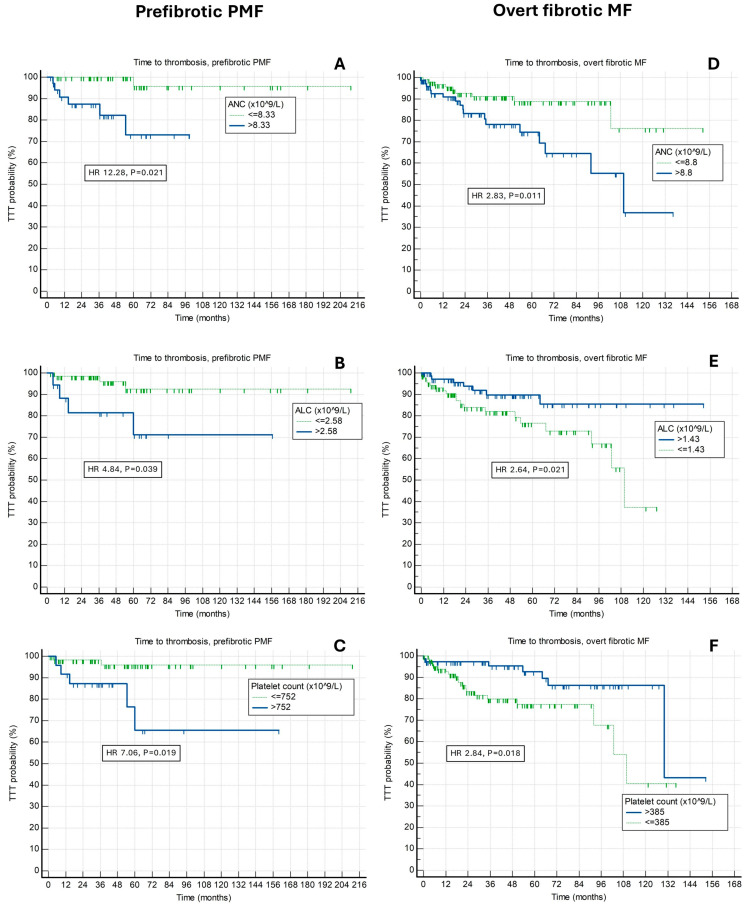
Associations of time to thrombosis (TTT) and (**A**) absolute neutrophil count (ANC), (**B**) absolute lymphocyte count (ALC) and (**C**) platelet count among prefibrotic primary myelofibrosis (PMF) patients, and (**D**) ANC, (**E**) ALC and (**F**) platelet count among overt fibrotic myelofibrosis patients.

When utilized for future thrombotic risk prediction in prefibrotic PMF patients at designated cut-off points, all higher ANC, higher ALC, higher platelet count, higher NLR and higher PLR had associations with shorter TTT for composite endpoint (*p* < 0.05 for all analyses). However, ANC > 8.33 had the best predictive properties (HR 12.28, *p* = 0.021, Harrel’s C 0.790) and encompassed substantial proportion of patients (35 [41.2%]). TTT curves for ANC, ALC and platelet count are shown in Figure 1A–C.

Considering arterial thrombotic events separately, higher ANC, higher ALC, higher platelet count and higher PLR (*p* < 0.05 for all analyses), but not NLR (*p* = 0.299) had significant associations with shorter TTT. Considering venous thrombotic events separately, higher ANC, higher ALC and higher NLR (*p* < 0.05 for all analyses), but not platelet count nor PLR (*p* > 0.05 for both analyses) had significant associations with shorter TTT. In both arterial and venous thrombotic contexts, ANC > 8.33 (HR 23.6, *p* = 0.011, Harrell’s C 0.840) for arterial and ANC > 14.19 (HR 11.29, *p* = 0.012, Harrell’s C 0.919) for venous thrombosis had the best prognostic properties among investigated indices.

We further analyzed the series of multivariate Cox regression models investigating the independent contribution of evaluated indices to composite thrombotic risk among prefibrotic PMF patients. ANC > 8.33 × 10^9^/L (HR 13.73, 95% CI [1.35–139.43], *p* = 0.026), ALC > 2.58 × 10^9^/L (HR 6.28, 95% CI [1.11–35.42], *p* = 0.037) and platelet count > 752 × 10^9^/L (HR 6.34, 95% CI [1.13–35.59], *p* = 0.035) were independently of each other associated with shorter TTT. Inclusion of NLR and PLR into the model rendered all investigated indices insignificant due to overadjustment/overlapping prognostic properties. Thus, ANC, ALC and platelet count were further evaluated in the final prognostic model that was additionally adjusted for age ≥60 years, history of thrombosis, male sex, JAK2 mutational status, presence of classic cardiovascular risk factors and chronic kidney disease, and which is presented in Table 4. In this model, ANC > 8.33 × 10^9^/L (HR 13.08, 95% CI [1.18–144.94], *p* = 0.036), ALC > 2.58 × 10^9^/L (HR 20.63, 95% CI [1.01–420.74], *p* = 0.049) and platelet count > 752 × 10^9^/L (HR 10.5, 95% CI [1.06–103.11], *p* = 0.043) remained independently associated with shorter TTT. The final prefibrotic PMF model had Harrell’s C index 0.930. Additional inclusion of use of cytoreductive therapy in synchronous analysis resulted in model overfitting and, when evaluated using the backwards model building approach, neither significantly contributed to prognostication of thrombotic risk, nor altered significance of ANC, ALC and platelet count, and was thus omitted from the final model. Due to insufficient statistical power, models for separate arterial and venous thromboses were not analyzed.

### 3.3. Relationship of Absolute Neutrophil, Absolute Lymphocyte, Platelet Count, Neutrophil-to-Lymphocyte and Platelet-to-Lymphocyte Ratios with Future Risk of Thrombosis in Overt Fibrotic MF Patients

Using the ROC curves analysis, specific cut-off points with best predictive properties for thrombotic risk prognostication for the context of overt fibrotic myelofibrosis were defined for ANC, ALC, platelets, NLR and PLR and are presented in Table 5. 

For prognostication of composite thrombotic endpoint, arterial and venous thromboses, respectively, the ROC curve-defined optimized cut-off values for ANC (×10^9^/L) were >8.8, >8.8, and no adequate cut-off, respectively, for ALC (×10^9^/L) were ≤1.43, no adequate cut-off, and no adequate cut-off, respectively, and for platelet count (×10^9^/L) were ≤385, ≤385, and no adequate cut-off, respectively. The ROC curve-defined cut-off values for NLR were >8, no adequate cut-off, and >9.67 respectively, and for PLR were no adequate cut-off, no adequate cut-off, and >397, respectively.

When utilized for future thrombotic risk prediction in overt fibrotic myelofibrosis patients at designated cut-off points, higher ANC, lower ALC, lower platelet count and higher NLR, but no PLR (*p* = 0.633) had significant associations with shorter TTT for composite endpoint (*p* < 0.05 for other analyses). All aforementioned indices had similar but overall modest prognostic properties and stratified patients into similarly sized groups encompassing around half of the overall cohort. TTT curves for ANC, ALC and platelet count are shown in Figure 1D–F.

Considering arterial thrombotic events separately, higher ANC and lower platelet count (*p* < 0.05 for both analyses), but not ALC, NLR nor PLR (*p* > 0.05 for all analyses) had significant associations with shorter TTT. Considering venous thrombotic events separately, higher NLR and higher PLR (*p* < 0.05 for both analyses) but not ANC, ALC nor platelet count (*p* > 0.05 for all analyses) had significant associations with shorter TTT.

We further analyzed the series of multivariate Cox regression models investigating independent contribution of evaluated indices to composite thrombotic risk among overt fibrotic MF patients. ANC > 8.8 × 10^9^/L (HR 3.81, 95% CI [1.61–8.98], *p* = 0.002), ALC ≤ 1.43 × 10^9^/L (HR 3.44, 95% CI [1.41–8.36], *p* = 0.006) and platelet count ≤ 385 × 10^9^/L (HR 2.91, 95% CI [1.13–7.48], *p* = 0.026) were independently of each other associated with shorter TTT. Inclusion of NLR and PLR into the model returned the same observation, with ANC, ALC and platelet count remaining significant, and NLR and PLR being insignificant when evaluated simultaneously. Thus, similarly as in the prefibrotic context, ANC, ALC and platelet count were further evaluated in the final prognostic model that was additionally adjusted for age ≥ 60 years, history of thrombosis, sex, JAK2 mutational status, presence of classic cardiovascular risk factors and chronic kidney disease, and which is presented in Table 4. In this model, ANC > 8.8 × 10^9^/L (HR 4.49, 95% CI [1.62–12.45], *p* = 0.004), ALC ≤ 1.43 × 10^9^/L (HR 4.15, 95% CI [1.65–10.47], *p* = 0.003), platelet count ≤ 385 × 10^9^/L (HR 4.68, 95% CI [1.61–13.66], *p* = 0.004) and chronic kidney disease (HR 9.07, 95% CI [3.26–25.18], *p* < 0.001) remained independently associated with shorter TTT. The final overt fibrotic myelofibrosis model had Harrell’s C index 0.805. Additional inclusion of use of cytoreductive therapy (in both synchronous analysis and via backwards model building approach) neither significantly added to prognostication of thrombotic risk nor altered significance of ANC, ALC, platelet count and chronic kidney disease with thrombotic outcome, and was thus omitted from the final model. Due to insufficient statistical power, models for separate arterial and venous thromboses were not analyzed.

**Table 1:** Patients’ characteristics among prefibrotic PMF patients, and their relationship to ANC, ALC and platelet count.

Prefibrotic PMF	Overall Characteristics	ANC	ALC	Platelet Count
Age (years)	66 IQR (56–73)	*p* = 0.008 * ↑	*p* = 0.432	*p* = 0.473
Male sex	57/85 (67.1%)	*p* = 0.771	*p* = 0.050 * ↓	*p* = 0.042 * ↑
BM fibrosis Grade 0 Grade I	13/85 (15.3%) 72/85 (84.7%)	*p* = 0.669	*p* = 0.802	*p* = 0.017 * ↑
*JAK2* mutated	57/84 (67.9%)	*p* = 0.022 * ↑	*p* = 0.081	*p* = 0.688
*CALR* mutated	10/64 (15.6%)	*p* = 0.002 * ↓	*p* = 0.882	*p* = 0.913
Constitutional symptoms	19/85 (22.4%)	*p* = 0.011 * ↑	*p* = 0.883	*p* = 0.044 * ↓
Transfusion dependency	10/85 (11.8%)	*p* = 1.000	*p* = 0.758	*p* = 0.037 * ↓
Massive splenomegaly	5/80 (6.3%)	*p* = 0.314	*p* = 0.432	*p* = 0.001 * ↓
Spleen size under left costal margin (cm)	0 IQR (0–4)	*p* = 0.213	*p* = 0.445	*p* < 0.001 * ↓
WBC (×10^9^/L)	10.9 IQR (8.4–16.4)	*p* < 0.001 * ↑	*p* < 0.001 * ↑	*p* = 0.758
Circulatory blasts ≥ 1%	17/85 (20%)	*p* = 0.668	*p* = 0.821	*p* < 0.001 * ↓
ANC (×10^9^/L)	7.5 IQR (5.5–12.08)	-	*p* = 0.002 * ↑	*p* = 0.687
ALC (×10^9^/L)	1.8 IQR (1.3–2.5)	*p* = 0.002 * ↑	-	*p* = 0.092
Abs. mono. (×10^9^/L)	0.6 IQR (0.4–0.91)	*p* < 0.001 * ↑	*p* < 0.001 * ↑	*p* = 0.054
Abs. basophils (×10^9^/L)	0.1 IQR (0.1–0.2)	*p* < 0.001 * ↑	*p* = 0.007 * ↑	*p* = 0.344
Hemoglobin (g/L)	132 IQR (117–148)	*p* = 0.689	*p* = 0.062	*p* = 0.054
Platelet count (×10^9^/L)	550 IQR (297–780)	*p* = 0.687	*p* = 0.092	-
LDH (U/L)	322 IQR (224.5–510.5)	*p* < 0.001 * ↑	*p* = 0.802	*p* = 0.106
CRP (mg/L)	1.9 IQR (0.8–5.4)	*p* = 0.085	*p* = 0.307	*p* = 0.007 * ↓
Albumin (g/L)	45 IQR (41.15–47.3)	*p* = 0.641	*p* = 0.903	*p* = 0.996
Uric acid (mmol/L)	380 IQR (301–450)	*p* = 0.001 * ↑	*p* = 0.730	*p* = 0.247
Charlson comorbidity index	3 IQR (2–4)	*p* = 0.032 * ↑	*p* = 0.927	*p* = 0.225
CV risk factors	60/83 (72.3%)	*p* = 0.538	*p* = 0.131	*p* = 0.348
Chronic kidney disease	15/82 (18.3%)	*p* = 0.035 * ↑	*p* = 0.648	*p* = 0.325
Arterial hypertension	49/83 (59%)	*p* = 0.752	*p* = 0.301	*p* = 0.876
Diabetes mellitus	12/83 (14.5%)	*p* = 0.835	*p* = 0.876	*p* = 0.478
Hyperlipoproteinemia	15/82 (18.3%)	*p* = 0.228	*p* = 0.536	*p* = 0.171
Obesity	9/62 (14.5%)	*p* = 0.771	*p* = 0.496	*p* = 0.335
Active smoking	10/72 (13.9%)	*p* = 0.980	*p* = 0.750	*p* = 0.352
History of thrombosis	15/85 (17.7%)	*p* = 0.828	*p* = 0.861	*p* = 0.071
Cytoreductive therapy	52/83 (62.7%)	*p* = 0.025 * ↑	*p* = 0.099	*p* = 0.006 * ↑
DIPSS (PMF) Low risk Intermediate-1 risk Intermediate-2 risk High risk	26/85 (30.6%) 40/85 (47.1%) 17/85 (20%) 2/85 (2.4%)	*p* = 0.412	*p* = 0.204	*p* = 0.071

* statistically significant at level *p* < 0.05. ↑ and ↓ signs depict direction of association. Abbreviations: PMF—primary myelofibrosis, ANC—absolute neutrophil count, ALC—absolute lymphocyte count, IQR—interquartile range, BM—bone marrow, JAK2—Janus kinase 2, CALR—Calreticulin, MPL—thrombopoietin receptor, Massive splenomegaly—palpable spleen ≥10 cm under the left costal margin, WBC—white blood cells, Abs.—absolute, LDH—lactate dehydrogenase, CRP—C reactive protein, CV—cardiovascular, DIPSS—Dynamic International Prognostic Scoring System.

**Table 2:** Patients’ characteristics among overt fibrotic myelofibrosis patients, and their relationship to ANC, ALC and platelet count.

Overt Fibrotic Myelofibrosis	Overall Characteristics	ANC	ALC	Platelet Count
Age (years)	68 IQR (61.5–76)	*p* = 0.223	*p* = 0.494	*p* = 0.185
Male sex	98/171 (57.3%)	*p* = 0.639	*p* = 0.578	*p* = 0.041 * ↑
Etiology of myelofibrosis PMF Post PV SMF Post ET SMF	93/171 (54.4%) 39/171 (22.8%) 38/171 (22.2%)	*p* = 0.055	*p* = 0.133	*p* = 0.116
BM fibrosis Grade II Grade II	113/171 (66.1%) 58/171 (33.9%)	*p* = 0.184	*p* = 0.892	*p* = 0.017 * ↓
*JAK2* mutated	120/164 (73.2%)	*p* = 0.002 * ↑	*p* = 0.898	*p* = 0.245
*CALR* mutated	12/135 (8.9%)	*p* = 0.103	*p* = 0.486	*p* = 0.052
*MPL* mutated	4/133 (3%)	*p* = 0.402	*p* = 0.668	*p* = 0.716
Constitutional symptoms	93/171 (54.4%)	*p* = 0.169	*p* = 0.394	*p* < 0.001 * ↓
Transfusion dependency	54/171 (31.6%)	*p* = 0.224	*p* = 0.407	*p* = 0.101
Massive splenomegaly	35/160 (21.9%)	*p* = 0.873	*p* = 0.041 * ↓	*p* = 0.150
Spleen size under left costal margin (cm)	4 IQR (1–10)	*p* = 0.200	*p* = 0.448	*p* = 0.007 * ↓
WBC (×10^9^/L)	10.4 IQR (6.05–19.15)	*p* < 0.001 * ↑	*p* < 0.001 * ↑	*p* < 0.001 * ↑
Circulatory blasts ≥ 1%	85/171 (49.7%)	*p* = 0.360	*p* = 0.002 * ↑	*p* = 0.073
ANC (×10^9^/L)	7.3 IQR (3.5–13.43)	-	*p* < 0.001 * ↑	*p* < 0.001 * ↑
ALC (×10^9^/L)	1.4 IQR (1–2.13)	*p* < 0.001 * ↑	-	*p* = 0.148
Abs. mono. (×10^9^/L)	0.4 IQR (0.21–0.8)	*p* < 0.001 * ↑	*p* < 0.001 * ↑	*p* < 0.001 * ↑
Abs. basophils (×10^9^/L)	0.1 IQR (0.06–0.3)	*p* < 0.001 * ↑	*p* < 0.001 * ↑	*p* = 0.022 * ↑
Hemoglobin level (g/L)	101 IQR (87.5–121.5)	*p* < 0.001 * ↑	*p* = 0.072	*p* = 0.002 * ↑
Platelets (×10^9^/L)	300 IQR (173–525.5)	*p* < 0.001 * ↑	*p* = 0.148	-
LDH (U/L)	485.5 IQR (343.25–729)	*p* = 0.008 * ↑	*p* = 0.002 * ↑	*p* = 0.667
CRP (mg/L)	6.7 IQR (2.3–13.4)	*p* = 0.931	*p* = 0.567	*p* < 0.001 * ↓
Albumin (g/L)	42 IQR (39–44.05)	*p* = 0.873	*p* = 0.368	*p* = 0.100
Uric acid (mmol/L)	381 IQR (318.75–467.25)	*p* = 0.004 * ↑	*p* = 0.014 * ↑	*p* = 0.018 * ↑
Charlson comorbidity index	3 IQR (2–4.25)	*p* = 0.258	*p* = 0.983	*p* = 0.910
CV risk factors	111/165 (67.3%)	*p* = 0.387	*p* = 0.209	*p* = 0.402
Chronic kidney disease	27/165 (16.4%)	*p* = 0.063	*p* = 0.601	*p* < 0.001 * ↑
Arterial hypertension	96/164 (58.5%)	*p* = 0.565	*p* = 0.661	*p* = 0.352
Diabetes mellitus	22/165 (13.3%)	*p* = 0.842	*p* = 0.641	*p* = 0.036 * ↓
Hyperlipoproteinemia	26/161 (16.1%)	*p* = 0.603	*p* = 0.104	*p* = 0.011 * ↑
Obesity	8/141 (5.7%)	*p* = 0.728	*p* = 0.731	*p* = 0.262
Active smoking	21/148 (14.2%)	*p* = 0.518	*p* = 0.149	*p* = 0.528
History of thrombosis	33/171 (19.3%)	*p* = 0.264	*p* = 0.615	*p* = 0.725
Cytoreductive therapy	116/167 (69.5%)	*p* < 0.001 * ↑	*p* = 0.394	*p* = 0.009 * ↑
DIPSS (PMF) Low risk Intermediate-1 risk Intermediate-2 risk High risk	4/93 (4.3%) 25/93 (26.9%) 52/93 (55.9%) 12/93 (12.9%)	*p* = 0.793	*p* = 0.094	*p* = 0.009 * ↓
MYSEC-PM (SMF) Low risk Intermediate-1 risk Intermediate-2 risk High risk	13/135 (9.6%) 42/135 (31.1%) 40/135 (29.6%) 40/135 (29.6%)	*p* = 0.165	*p* = 0.208	*p* = 0.005 * ↓

* statistically significant at level *p* < 0.05. ↑ and ↓ signs depict direction of association. Abbreviations: ANC—absolute neutrophil count, ALC—absolute lymphocyte count, IQR—interquartile range, PMF—primary myelofibrosis, PV—polycythemia vera, SMF—secondary myelofibrosis, ET—essential thrombocythemia, BM—bone marrow, JAK2—Janus kinase 2, CALR—Calreticulin, MPL—thrombopoietin receptor, Massive splenomegaly—palpable spleen ≥10 cm under the left costal margin, WBC—white blood cells, Abs.—absolute, LDH—lactate dehydrogenase, CRP—C reactive protein, CV—cardiovascular, DIPSS—Dynamic International Prognostic Scoring System, MYSEC-PM—myelofibrosis secondary to PV and ET prognostic model.

**Table 3:** Overview of specific cutoffs for hematological indices and their prognostic properties among prefibrotic PMF patients.

Prefibrotic PMF	Time to Thrombosis (Composite)	Time to Arterial Thrombosis	Time to Venous Thrombosis
ANC (×10^9^/L) ROCc defined cut-off Proportion of patients Associated risk Harrell’s C	>8.33 35 (41.2%) HR 12.28, *p* = 0.021 * 0.790	>8.33 35 (41.2%) HR 23.6, *p* = 0.011 * 0.840	>14.19 15 (17.6%) HR 11.29, *p* = 0.012 * 0.919
ALC (×10^9^/L) ROCc defined cut-off Proportion of patients Associated risk Harrell’s C	>2.58 19 (22%) HR 4.84, *p* = 0.039 * 0.703	>4 3 (3.5%) HR -, *p* < 0.001 * 0.728	>2.6 18 (21.2%) HR 21.28, *p* = 0.036 * 0.739
Platelet count (×10^9^/L) ROCc defined cut-off Proportion of patients Associated risk Harrell’s C	>752 25 (28.4%) HR 7.06, *p* = 0.019 * 0.709	>574 41 (46.6%) HR 14.9, *p* = 0.029 * 0.799	Not adequate > 752 - *p* = 0.156 0.682
NLR ROCc defined cut-off Proportion of patients Associated risk Harrell’s C	>6.33 24 (28.2%) HR 4.87, *p* = 0.040 * 0.726	Not adequate > 4.86 - *p* = 0.299 0.583	>6.33 24 (28.2%) HR 35.5, *p* = 0.005 * 0.871
PLR ROCc defined cut-off Proportion of patients Associated risk Harrell’s C	>498 13 (15.3%) HR 5.41, *p* = 0.027 * 0.634	>498 13 (15.3%) HR 14.92, *p* = 0.027 * 0.718	Not adequate ≤ 291 - *p* = 0.357 0.631

* statistically significant at level *p* < 0.05. Abbreviations: PMF—primary myelofibrosis, ANC—absolute neutrophil count, ROCc—receiver operating characteristic curve, HR—hazard ratio, ALC absolute lymphocyte count, NLR—neutrophil-to-lymphocyte ratio, PLR—platelet-to-lymphocyte ratio.

**Table 4:** Multivariate Cox regression models for prediction of thrombotic risk for composite arterial and venous thrombotic outcome among prefibrotic and overt fibrotic myelofibrosis patients.

Variables	Prefibrotic PMF	Overt Fibrotic Myelofibrosis
ANC (×10^9^/L)	Cut-off >8.33; *p* = 0.036 * HR 13.08, 95% CI (1.18–144.94)	Cut-off >8.8; *p* = 0.004 * HR 4.49, 95% CI (1.62–12.45)
ALC(×10^9^/L)	Cut-off >2.58; *p* = 0.049 * HR 20.63, 95% CI (1.01–420.74)	Cut-off ≤1.43; *p* = 0.003 * HR 4.15, 95% CI (1.65–10.47)
Platelet count (×10^9^/L)	Cut-off >752; *p* = 0.043 * HR 10.5, 95% CI (1.07–103.11)	Cut-off ≤385; *p* = 0.004 * HR 4.68, 95% CI (1.61–13.66)
Age ≥ 60 years	*p* = 0.184 HR 6.94, 95% CI (0.39–121.71)	*p* = 0.846 HR 1.11, 95% CI (0.36–3.44)
History of thrombosis	*p* = 0.431 HR 0.17, 95% CI (0.0–13.41)	*p* = 0.231 HR 1.81, 95% CI (0.68–4.80)
Male sex	*p* = 0.441 HR 3.11, 95% CI (0.17–56.23)	*p* = 0.477 HR 0.72, 95% CI (0.29–1.77)
JAK2 mutated	*p* = 0.735 HR 1.48, 95% CI (0.14–14.75)	*p* = 0.919 HR 1.06, 95% CI (0.30–3.68)
Classic CV risk factors	*p* = 0.491 HR 2.82, 95% CI (0.14–54.62)	*p* = 0.747 HR 1.16, 95% CI (0.45–2.96)
CKD	*p* = 0.569 HR 1.9, 95% CI (0.21–17.41)	*p* < 0.001 * HR 9.07, 95% CI (3.26–25.18)

* statistically significant at level *p* < 0.05. Abbreviations: PMF—primary myelofibrosis, ANC—absolute neutrophil count, HR—hazard ratio, CI—confidence interval, ALC—absolute lymphocyte count, JAK2—Janus kinase 2, CV—cardiovascular, CKD—chronic kidney disease.

**Table 5:** Overview of specific cutoffs for hematological indices and their prognostic properties among overt fibrotic myelofibrosis patients.

Overt Fibrotic Myelofibrosis	Time to Thrombosis (Composite)	Time to Arterial Thrombosis	Time to Venous Thrombosis
ANC (×10^9^/L) ROCc defined cut-off Proportion of patients Associated risk Harrell’s C	>8.8 75 (43.9%) HR 2.83, *p* = 0.011 * 0.612	>8.8 75 (43.9%) HR 2.43, *p* = 0.048 * 0.579	Not adequate < 9.9 - *p* = 0.223 0.619
ALC (×10^9^/L) ROCc defined cut-off Proportion of patients Associated risk Harrell’s C	≤1.43 90 (52.6%) HR 2.64, *p* = 0.021 * 0.604	Not adequate ≤ 1.5 - *p* = 0.142 0.562	Not adequate ≤ 1.43 - *p* = 0.112 0.664
Platelet count (×10^9^/L) ROCc defined cut-off Proportion of patients Associated risk Harrell’s C	≤385 105 (58%) HR 2.84, *p* = 0.018 * 0.624	≤385 105 (58%) HR 4.32, *p* = 0.009 * 0.651	Not adequate > 226 - *p* = 0.282 0.559
NLR ROCc defined cut-off Proportion of patients Associated risk Harrell’s C	>8 46 (26.9%) HR 2.68, *p* = 0.012 * 0.592	Not adequate > 8 46 (26.9%) *p* = 0.150 0.535	>9.67 37 (21.6%) HR 6.53, *p* = 0.015 * 0.708
PLR ROCc defined cut-off Proportion of patients Associated risk Harrell’s C	Not adequate ≤ 477 - *p* = 0.633 0.508	Not adequate ≤ 477 - *p* = 0.129 0.577	>397 42 (24.6%) HR 4.1, *p* = 0.070 0.659

* statistically significant at level *p* < 0.05. Abbreviations: ANC—absolute neutrophil count, ROCc—receiver operating characteristic curve, HR—hazard ratio, ALC absolute lymphocyte count, NLR—neutrophil-to-lymphocyte ratio, PLR—platelet-to-lymphocyte ratio.

## 4. Discussion

To the best of our knowledge, the current study is the first to systematically evaluate utility of ANC, ALC, platelet count and their ratios, NLR and PLR, for thrombotic risk stratification in patients with prefibrotic and overt fibrotic MF. Current data, as well as previous reports [3,4,8], confirm the high thrombotic risk associated with the MPN phenotype of MF, which seems to be more pronounced in overt fibrotic MF patients. Thrombosis imposes a substantial morbidity burden on MF patients [42,43] and mandates the introduction of specific therapies that may further make it difficult to optimize care [44]. Myelodepletive phenotype, low platelet count and developed complications of portal hypertension that are encountered in subsets of MF patients further challenge the delivery of specific primary and secondary prevention measures [45,46,47,48,49,50]. Thrombosis also significantly increases healthcare resource utilization and treatment costs [51], which are already high in patients with MF [52]. The consequent impairment of functional status associated with thrombotic events may further impact eligibility to some anticancer therapies and push the patient into the sphere of palliative care. Nevertheless, thrombotic risk in MF patients is of secondary interest due to anemia, constitutional symptoms and the risk of death dominating the care environment [2,47,53,54]. As such, there are no official guidelines of prognostic risk factors used for this purpose among MF patients, with notable exception of the original International Prognostic Score of Thrombosis for Essential Thrombocythemia (IPSET-Thrombosis) score, originally developed for ET, being validated among prefibrotic PMF patients [55]. However, several clinical characteristics have been shown to bear higher thrombotic risk in MF patients, including leukocytosis [56], chronic kidney disease [57], higher estimated plasma volume status [58] and post-PV etiology of MF [8].

CBC indices and derived measures have been widely investigated in various cardiovascular, inflammatory and oncologic contexts [59], resulting in consistently present prognostic potential of these measures. Although highly non-specific and prone to inter and intra variability, as well as to different confounders, these parameters that are indicative of the quality of hematopoiesis seem to be highly responsive to even subclinical inflammatory and nutritive deflections [60]. Among MPN patients, red blood cell indices, relative lymphocyte count, ANC, ALC, NLR and PLR have been associated with poor prognosis, although in different directions specific to investigated contexts [32,33,34,61,62,63,64,65]. This is probably mostly due to the impact of the different biology of investigated MPN subsets, as well as to the specific impact of particular therapies. For example, although higher red blood cell distribution width (RDW) has been associated with higher thrombotic risk in patients from the general population, as well as in patients with PV and MF [61,62,63], machine learning (ML)-based analysis of the OPTUM database recognized lower RDW to be predictive of thrombotic events among PV patients treated with hydroxyurea [64], potentially implicating non-adherence or non-responsiveness to this type of treatment that is known to elevate RDW. In contrast to the non-MPN population, among MPN patients it is very hard to decipher to what extent deflections in CBC indices may represent proliferative potential of myeloproliferative disease itself or inflammatory-driven responses [32]. This is the particular reason why comprehensive evaluation of these indices in patients with MF is needed, and why we undertook the current study.

As our data show, ALC, platelet count and PLR significantly differed regarding the degree of BM fibrosis. In both prefibrotic and overt fibrotic contexts, higher ANC was associated with JAK2 mutation, higher uric acid levels and increase in other WBC subsets, indicative of higher myeloproliferative drive. The number of associations of ANC with features implying the stronger myeloproliferative drive was higher in overt fibrotic context (higher LDH, hemoglobin, and platelet count). Despite a similar positive relationship with other WBC subsets, ALC did not consistently show associations with other specific disease features. Higher platelet count was associated with male sex, absence of constitutional symptoms, less advanced spleen size and lower CRP in both contexts. However, platelet count was associated with higher degree of BM fibrosis among prefibrotic and lower degree of BM fibrosis and presence of constitutional symptoms among overt fibrotic MF patients. Higher platelet count was indicative of preserved bone marrow hematopoietic potential (higher hemoglobin and higher WBC) among overt fibrotic MF patients, which was not evident among prefibrotic patients. 

Considering thrombotic risk stratification, higher ANC was associated with higher risk of thrombosis for both prefibrotic and overt fibrotic MF patients, whereas different directions of ALC and platelet count were observed: prefibrotic patients having poor prognosis associated with elevated ALC and platelet count and overt fibrotic patients having poor prognosis associated with lower ALC and lower platelet count. This probably reflects stronger proliferative potential of the disease among prefibrotic patients and more advanced features and features of myelodepletive phenotype in overt fibrotic patients. ANC, ALC and platelet count at designated cut-off levels were able to predict higher thrombotic risk independently of each other, both in prefibrotic and overt fibrotic patients. Moreover, they had strong prognostic potential to overshadow other variables usually associated with higher thrombotic risk in MPN patients, with notable exception of chronic kidney disease, which was able to independently contribute to prognostic risk stratification based on these hematological indices among overt fibrotic patients. NLR and PLR were not able to provide better prognostic information than those given by individual counts. This is likely to occur due to the association of higher ALC with inferior outcome in prefibrotic patients, resulting in diminished properties of higher ANC and platelet count when adjusted for ALC in respective ratios. It should also be noted that the combination of ANC, ALC and platelet count provided very accurate prognostic discrimination in both contexts of prefibrotic and overt fibrotic MF (correctly stratifying 93% and 81% of patients developing thrombotic event, respectively). Such high prognostic accuracy is likely the result of using the same cohort for definition and evaluation of specific cut-off levels, mandating further validation in independent datasets. Taken together, CBC indices seem to be highly attractive as simple, instantly available and potent predictors of thrombotic risk in MF patients. It should be noted, however, that no universally applicable cut-off levels for thrombotic risk stratification seem to be possible in all MPN patients, even in patients with similar disease at different levels of BM fibrosis development.

The limitations of the current study are its retrospective design and inability to control various unmeasured variables potentially affecting thrombotic risk. No causality can be inferred from presented associations due to limitations of the study design. Thus, both predictors and outcomes, as well as third unmeasured variables, may be the underlying cause of the given observations. In addition, the small numbers of patients and events in specific subgroups limit possible insights into associations of particular indices and outcomes, due to the loss of statistical power. Due to the risk of immortalization bias associated with the analysis of specific therapies introduced during later time points, we could not reliably evaluate the contribution of cytoreductive and other specific therapies that patients were exposed to post baseline (specific JAK inhibitors approved during study period, stem cell transplantation, etc.), and only baseline use of cytoreductive therapy was analyzed. The strength of the current study is its systematic overview of the relationship of ANC, ALC, platelet count, NLR and PLR with different aspects of thrombotic risk in MF patients, stratified regarding the degree of BM fibrosis. Current observations may aid in better understanding the complex relationship between specific blood components in patients with prefibrotic and overt fibrotic MF, as well as guide the development of novel prognostic scores in patients with MF.

## 5. Conclusions

In patients with both prefibrotic and overt fibrotic MF, prognostic properties of ANC, ALC and platelet count are mutually independent and exceed those of NLR and PLR regarding thrombotic risk stratification. Higher ANC is worse in both contexts, but ALC and platelet count differ in the direction of association with thrombotic risk in prefibrotic and overt fibrotic MF patients.

## Data Availability

Data are available from the corresponding author on reasonable request.
